# Comparisons of Visceral Adiposity Index, Body Shape Index, Body Mass Index and Waist Circumference and Their Associations with Diabetes Mellitus in Adults

**DOI:** 10.3390/nu11071580

**Published:** 2019-07-12

**Authors:** Junxiang Wei, Xin Liu, Hong Xue, Youfa Wang, Zumin Shi

**Affiliations:** 1 Department of Epidemiology and Biostatistics, School of Public Health, Xi’an Jiaotong University Health Science Center, Xi’an, Shaanxi 710061, China; 2 Global Health Institute, Xi’an Jiaotong University Health Science Center, Xi’an, Shaanxi 710061, China; 3 Department of Health Behavior and Policy, School of Medicine, Virginia Commonwealth University, Richmond, VA 23298, USA; 4 Human Nutrition Department, Qatar University, Doha 2713, Qatar

**Keywords:** obesity, visceral adiposity index, body shape index, waist circumference, body mass index, diabetes mellitus

## Abstract

The associations between visceral adiposity index (VAI), body shape index and diabetes in adults were inconsistent. We assessed the predictive capacity of VAI and body shape index for diabetes by comparing them with body mass index (BMI) and waist circumference (WC). We used the data of 5838 Chinese men and women aged ≥18 years from the 2009 China Health and Nutrition Survey. Multivariate logistic regression analysis was performed to examine the independent associations between Chinese VAI (CVAI) or body shape index and diabetes. The predictive power of the two indices was assessed using the receiver-operating characteristic (ROC) curve analysis, and compared with those of BMI and WC. Both CVAI and body shape index were positively associated with diabetes. The odds ratios for diabetes were 4.9 (2.9–8.1) and 1.8 (1.2–2.8) in men, and 14.2 (5.3–38.2) and 2.0 (1.3–3.1) in women for the highest quartile of CVAI and body shape index, respectively. The area under the ROC (AUC) and Youden index for CVAI was the highest among all four obesity indicators, whereas BMI and WC are better indicators for diabetes screening. Higher CVAI and body shape index scores are independently associated with diabetes risk. CVAI has a higher overall diabetes diagnostic ability than BMI, WC and body shape index in Chinese adults. BMI and WC, however, are more appealing as screening indicators considering their easy use.

## 1. Introduction

Diabetes mellitus (DM) has become a major worldwide public health burden in the past decade, especially in developing countries [1]. The China national Diabetes and Metabolic Disorders Study reported prevalence of diabetes was 9.7% among adults in 2010 [2,3]. This increased trend of DM is concurrent with rising rate of obesity in China, and excessive body fat has been proven to be a crucial pathogenic factor for insulin resistance [4]. Excessive body fat disposed in the ectopic tissue, such as visceral adiposity tissue (VAT), may cause dysfunctional adiposity and it plays a vicious role in metabolic diseases [5]. In addition, body fat distribution is related to metabolic disturbances and metabolic disorders [6]. Thus, knowing the ability to predict the visceral adiposity index for diabetes risk is greatly needed.

Body mass index (BMI), widely used since the early 1990s worldwide for classifying overweight and obesity, as well as studying obesity associated risks, provides reliable information concerning body weight excess, but does not differentiate fat from lean mass [7,8,9]. Waist circumference (WC) is a simple anthropometric parameter for abdominal adiposity and it reflects visceral obesity better than BMI [10]. It is a better indicator of obesity associated risks for DM, as shown by our research [11,12], though it has limitations in distinguishing VAT from subcutaneous fat mass [13]. The magnetic resonance imaging (MRI) and computed tomography (CT) are considered the gold standard for body fat determination, but they were less recommended in routine clinical practice due to their unavailability.

The VAI, which is comprised of anthropometric measures like BMI, WC and clinical measures of serum triglycerides (TG) and high-density lipoprotein-cholesterol (HDL-C) levels, was shown to be a better surrogate index than these single anthropometric indices in predicting insulin resistant-related metabolic disorders [14,15]. However, evaluating the predicting performance of VAI, which was developed for Caucasians, on people who have diabetes in Chinese population may lead to inaccurate results [16,17]. Meanwhile, a body shape index (ABSI), which encompasses waist circumference and BMI, particularly depicts fat distribution [18,19], and was shown to be a reliable index of body fat accumulation. However, there is little research on this in China [20].

Therefore, we hypothesize that non-invasive, clinically measurable surrogates could be useful in identifying body fat distribution and help predict diabetes risk. We aim to examine the associations between these two indicators and diabetes risk, and to investigate their performance in identifying diabetes compared with BMI and WC in Chinese adults.

## 2. Materials and Methods

### 2.1. Study Design and Population

Data collected in the 2009 China Health and Nutrition Survey (CHNS) was used in this study. The CHNS is an ongoing, open, multipurpose household based cohort study since 1989. It adapts a multistage random-cluster sampling process and is conducted in 9 provinces (Liaoning, Heilongjiang, Shandong, Jiangsu, Henan, Hubei, Hunan, Guangxi and Guizhou), which covers approximately 56% of China’s population. It was designed to represent a large set of the population, varying significantly in geography, economic development, public resources and health status. More detailed information about the CHNS has been described elsewhere [21]. The CHNS was approved by the institutional review committee of the University of North Carolina at Chapel Hill and Chinese Center for Disease Control and Prevention. All participants were required to provide written informed consents before their participation.

Using CHNS data, we assessed the associations between the two indicators and diabetes risk, since fasting blood samples were collected initially in 2009 and the biochemical data collected in 2015 is unavailable currently. A structured questionnaire was used to obtain information on sex, age and lifestyle behaviors. Participants aged ≥18 years and without missing information on physical examination and biochemical measurements were included. Exclusion criteria included pregnancy and no information on age, sex and lifestyle behavior indicators. Ultimately, a total number of 5838 adults with anthropometry and clinical examination information were included in the analysis (Figure S1).

### 2.2. Definition of Key Study Outcome DM

DM was defined as having FPG ≥ 7.0 mmol/L, HbA1c ≥ 6.5%, previous diagnosis of DM, or use of antidiabetic medications. Of the 5838 subjects, 482 (8.3%) had DM.

### 2.3. Anthropometry and Biochemical Measurements

Weight (kg) and height (m) were measured according to standard methods, and body mass index (BMI) was calculated as weight/height squared (kg/m^2^). WC was measured to the nearest 0.1 cm at the middle point between the bottom of the rib cage and the uppermost border of the iliac crests at the end of exhalation in standing positions with an inelastic tape. Standard mercury sphygmomanometers were used to measure blood pressure by trained investigator at three different consecutive times at 3–5 min intervals on one visit. All physical examinations were performed following the same protocol at each study visit and study site.

### 2.4. Biochemical Measurements

Blood was collected from the participants after an at least 8 h overnight fast. The whole blood was centrifuged immediately after collection, and plasma and serum samples were then frozen and stored at −87 °C for future analysis. Samples for fasting plasma glucose (FPG) and glycated hemoglobin (HbA1c) measurements were tested immediately. Serum glucose was measured by the glucose oxidase phenol 4-aminoantipyrine peroxidase (GOD-PAP) methods with a Hitachi 7600 analyzer (Hitachi, Tokyo, Japan). Whole blood HbA1c was measured with a high-performance liquid chromatography system (model HLC-723 G7, Tosoh Corporation, Tokyo, Japan). Fasting insulin concentration was tested using the radioimmunology assay (Gamma counter XH-6020, Xi’an, China). Lipids including total cholesterol (TC), Total triglycerides (TG), low-density lipoprotein cholesterol (LDL-C) and HDL-C, as well as uric acid, were measured using a biochemical auto-analyzer (Hitachi 7600 automated analyzer, Tokyo, Japan). Hypersensitive C-reactive protein (hs-CRP) was determined by the immunoturbidimetric method.

The VAI score was calculated using the specific formula for Chinese population [22]:

Males: CVAI = −267.93 + 0.68 × age + 0.03 × BMI + 4.00 × WC + 22.00 × log10(TG) − 16.32 × HDL

Females: CVAI = −187.32 + 1.71 × age + 4.23 × BMI + 1.12 × WC + 39.76 × log10(TG) − 11.66 × HDL.

ABSI was calculated as WC/(BMI^2/3^ × height^1/2^) and expressed in m^11/6^kg^−2/3^ [19]. The homeostasis model assessment of insulin resistance (HOMA-IR) was estimated as HOMA-IR = Fasting glucose (mmol/L) × fasting insulin (μIU/mL)/22.5 [23].

### 2.5. Statistical Analysis

Descriptive analyses were presented on the basis of gender-specific quartiles of CVAI and ABSI scores in order to control for the well-known sexual dimorphism in body composition. The characteristics were presented as mean (SD) and median with interquartile range (25–75%) given in parentheses for normalized continuous and skewed variables. Categorical variables were expressed as numbers and percentages. One-way analysis of variance (ANOVA) or the Mann–Whitney U-test was used for comparisons of quantitative variables among groups. Chi-squared test was performed to assess differences in proportions across groups. Partial correlations between two anthropometric indices and metabolic parameters adjusting for age and sex were evaluated with Pearson’s/Spearman’s correlation analysis.

Multivariable logistic regression models were performed to estimate the odds ratios (ORs) and 95% confident interval (95% CI) of DM associated with these two indices in four models for men and women, respectively. Receiver operating characteristic (ROC) curve analyses were used to compare the diagnostic performance of CVAI and ABSI as compared with BMI and WC for DM risk. A user written command *cutpt* was used to calculate the Youden index [24,25].

All statistical analyses were conducted using Stata software (version 15.0). A two-tailed statistical measure was used with a p-value of less than 0.05 is considered significant.

## 3. Results

### 3.1. Characteristics of the Study Population Classified According to the CVAI Quartiles

Subject characteristics are shown in Table 1. For both men and women, there were significant dose-response relationships of CVAI with all variables including clinical indicators and anthropometry indices (*p* < 0.001), with the exception of current drinking. A higher proportion of current smoking was found in women with higher CVAI, whereas the proportion decreased in men. HDL-C was inversely associated with elevated CVAI scores in men and women (both *p* < 0.001). The proportion of both men and women increased progressively with increasing CVAI scores.

### 3.2. Characteristics of the Study Population Grouped by ABSI Levels

In both genders, subjects with higher ABSI presented with higher waist circumference, blood pressure, fasting blood glucose and HbA1c (all *p* < 0.001). There were no significant differences for HDL-C, blood insulin and the percentages of current smoking and drinking among ABSI quartile groups in both men and women. Age, BMI, LDL-C and uric acid were gradually increased across the quartiles in women only. Both men and women had the increased prevalence of diabetes with increasing quartiles of ABSI scores (Table 2).

### 3.3. Partial Correlation Analysis with Metabolic Variables

Table 3 shows CVAI positively correlated with FPG, blood insulin, HbA1c, lipid profiles and uric acid (all *p* < 0.001), and negatively with HDL-C after adjusting for age and sex. The correlation between ASBI and the metabolic indicators showed similar patterns, but the correlation coefficients were smaller than correlation with CVAI.

### 3.4. ORs of CVAI or ABSI with Risk of Diabetes

Multivariable logistic regression models showed that the ORs for diabetes increased with elevated quartiles of the CVAI or ABSI score for both men and women in all four models (Table 4). The independent association of CVAI or ABSI with diabetes was stronger in women than in men, although the cut-point for CVAI and ABSI quartiles was higher in men than in women. The age-adjusted associations (OR, 95% CI) with diabetes for the second, third and fourth CVAI quartiles in men were 2.0 (1.2–3.5), 2.6 (1.5–4.3) and 5.9 (3.6–9.6), in comparison with the first quartile (Model 1). The corresponding figures were 3.6 (1.3–9.7), 10.5 (4.1–27.1) and 22.6 (8.7–58.9) for women, respectively. Significant associations were found for diabetes with the third and fourth ABSI quartiles in all subjects. These association estimators were basically unchanged after additionally adjusting for lifestyle behavior factors and economic status (Model 2). After additional adjusting for blood pressure and inflammatory biomarkers, the OR increased by about 10% (Model 3 and Model 4).

### 3.5. Receiver-Operating Characteristic (ROC) Curve Analysis and Optimum Thresholds for Anthropometric Indices

**Figure 1:**
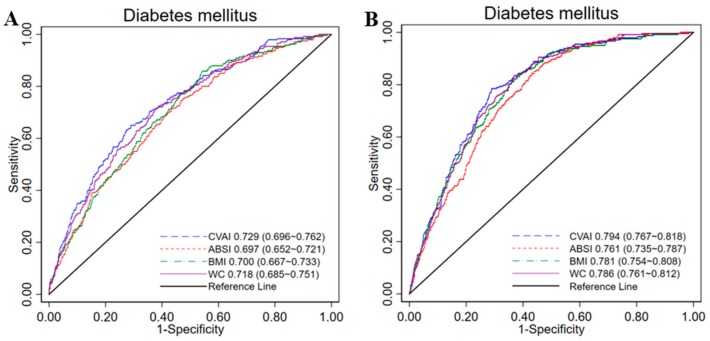
The ROC curves of CVAI, ABSI, BMI and WC for diabetes among men (**A**) and women (**B**) in China. ROC, receiver-operating characteristic; CVAI, Chinese visceral adiposity index; ABSI, a body shape index; BMI, body mass index; WC, waist circumference; AUCs, area under curves. AUCs were 0.729 and 0.794 for diagnosis of diabetes for men and women, respectively, and this is significantly better than ABSI, BMI and WC in Chinese adults (All *p* < 0.01).

Among all the 4 anthropometric indices, CVAI had the highest AUC values for diabetes in men (AUC = 0.729, 95% CI 0.696–0.762) and in women (AUC = 0.794, 95% CI 0.767–0.818). ABSI had the lowest AUC values in both sexes (men: AUC 0.679, 95% CI 0.552–0.721; women: AUC 0.761, 95% CI 0.735–0.787). The diagnostic performance of CVAI is similar with waist circumference in Chinese subjects (Figure 1). Table 5 shows the sensitivity, specificity and corresponding optimal cut-off values of each index for identifying diabetes by gender. CVAI had the highest Youden index values for identifying diabetes in men (0.36) and in women (0.50); the optimal CVAI cut-off was 107.27 in men and 88.15 in women.

## 4. Discussion

This study assessed the associations of the Chinese visceral adiposity index (CVAI) and body shape index (ABSI)—two indicators of adiposity distribution and function—with DM risks among adult Chinese people. We found graded positive associations of CVAI and ABSI scores with diabetes risks in both men and women. Partial correlation analysis found that the CVAI and ABSI were independently related to insulin resistance and lipid profiles. We also showed that the CVAI is superior to ABSI, BMI and waist circumference in predicting diabetes in both genders.

Increase in the prevalence of obesity and physical inactivity was concurrent with a tripling in diabetes incidence and other metabolic diseases over the past few decades in China [26]. Of the many obesity phenotypes, visceral obesity/fat is more metabolically deleterious than general obesity or subcutaneous fat, and it has been proposed as a marker of adiposity dysfunctional and ectopic fat deposition, which in turn leads to lipotoxicity and insulin resistance. Given that quantitating visceral adipose using CT or MRI is not feasible in large cohort studies and daily investigation, some simple clinical anthropometric indices such as WC and WHtR were used as surrogate indices of visceral adiposity to identify metabolic diseases. However, these classic anthropometric indices have the common shortcoming of their inability to take metabolic measures into consideration, and thus are not robust in various populations.

The present study adopted a developed indicator of visceral adiposity for the Chinese population, which is reliable for evaluating metabolic risk [27] and highest discriminatory power for dysglycemia in male and females. Consistent with other studies [22,28], our results showed that the CVAI score is associated with diabetes risk and is a good clinical index for prediction of visceral fat dysfunction. The associations of obesity indicators, including BMI and WC, with incident diabetes in multiethnic populations worldwide have been confirmed by several meta-analysis studies by the significant pooled estimates of the relative risk [29,30]. In our study, the CVAI score was highly correlated to and behaved slightly better in diabetes prediction than BMI and WC in Chinese adults by means of a higher AUC and an overall differentiating ability. Thus, CVAI is a useful clinical obesity indictor for diabetes risk when biospecimen data are available.

Previously, ABSI was developed base on the US National Health and Nutrition Examination Survey (NHANES) 1999–2004 data, which included several ethnicities like Mexican, other Hispanic, white, black, or other. For a given height and weight, ABSI is suitable for reflecting VAT [31]; however, subsequent research revealed conflicting results regarding its predictability for chronic diseases and mortality [31,32,33]. A study conducted in Dutch adults showed that ABSI was not a suitable index to identify CVD or CVD risk factors [34]. ABSI was not a better predictor of diabetes, hypertension or dyslipidaemia than WC or BMI in Japanese adults [35]. By contrast, Zhao and colleagues [18] recently indicated that ABSI had a better predictive ability than BMI in predicting diabetes in Han Chinese people in Northeast China. In agreement with previous studies [31,36,37], our study indicates that ABSI may not be a better predictor of diabetes than BMI or WC.

Furthermore, we incorporate types of staple foods into the analysis to examine the reason of conflicting findings with Zhao et al. People living the North China mainly consume wheat products. After re-analyzed the ROC separately by rice/wheat intake, our results showed that ABSI had a better predictive ability than BMI in men whose staple food is rice (results not reported), which is consistent with Zhao’s findings that ABSI is better than BMI in predicting diabetes in the Northeast Chinese population.

Interestingly, our study showed that increasing CVAI scores associated stronger with diabetes in women than men, whereas the ABSI had comparable predicting of diabetes risk in men and women. The explanations for these issues remain to be elucidated, and this may be related to gender differences in patterns of visceral fat deposition and regional adipose tissue distribution [38]. The mechanisms by which visceral adiposity can lead to diabetes may be different in men versus women. Besides, age is a well-established risk factor for diabetes, and women in the 4th CVAI quartile had a higher age than men in this group, hence diabetes risk may be partly explained by age and other related unfavorable traits. We think CVAI and ABSI serve as markers of diabetes risk for both men and women, but women should pay more attention to their visceral fat deposition. 

Our results showed that both CVAI and ABSI indicate close correlations with lipid levels, insulin resistance and inflammation in both men and women (*p* < 0.01). In addition, all four anthropometric indices exhibited the capability to identify individuals with diabetes (all AUC > 0.5 for all), and CVAI together with ABSI are strong and independent risk factors for diabetes (all ORs > 1). However, CVAI demonstrated the strongest prediction ability (AUC = 0.729 in men and 0.794 in women) among all anthropometric indices and ABSI had the weakest association with diabetes risk in both men and women. This might be because CVAI serve as a strong surrogate marker of visceral adiposity dysfunction, while ABSI, similar to BMI and WC, does not differentiate excess central adiposity in Chinese adults. Despite a high AUC value, CVAI has the lowest sensitivity among all the obesity indicators in both genders. It might not be the best indicator for the screening of diabetes.

The cutoffs for BMI and WC in predicting risk of T2DM in Chinese adults remain controversial. In response to WHO’s recommendations on the cutoff values for public health action for Asians, the Working Group on Obesity in China (WGOC) suggested that BMI ≥ 24 signifies overweight in Chinese population [39]. In the present study, the optimal cut-off values for Chinese men and women were found to be approximately BMI = 25 and 23, and 88.0 and 85.0 cm for WC, respectively. The current recommended cutoffs regarding central obesity in China are WC ≥ 90 cm for men and WC ≥ 90 for women. Compared with WGOC definitions and WC recommendations, our data observed a similar cutoff value for WC in women, but a lower value in men, and a sex difference for BMI. However, these results need be interpreted with caution because these cutoffs were based on data collected in a sample of 2433 men and 3320 women aged 52 ± 14 from 9 provinces in China.

Our study has several limitations. First, the cross-sectional data analyses cannot make causal inferences regarding the relationships between CVAI or ABSI and DM risks. Second, no direct measure of insulin resistance was done, and we were unable to directly assess the association of CVAI/ABSI with insulin resistance. Third, data on 2 h postprandial glucose was unavailable, which might lead to the underdiagnoses of some diabetic subjects. Key strengths of the study include that the data set provide rich related measures and a large sample allows us to assess the various associations and the predicting power of a set of indices.

## 5. Conclusions

In conclusion, our study indicated that both CVAI and ABSI are strong and independent risk factors for diabetes among Chinese adults. Superior to that of the BMI, WC and ABSI, CVAI demonstrates the best predictive power for metabolic disorders based on the Youden index in both genders. However, for the diabetes screening purpose, BMI and WC are better indicators than CVAI considering their higher sensitivity and accessibility and easy use in different settings, including by the public.

## Figures and Tables

**Table 1:** Demographic and clinical characteristics of study participants (*n* = 5838) across Chinese visceral adiposity index (CVAI) quartiles in China.

	Men					Women				
	Q1(<57)	Q2(57–89)	Q3(89–122)	Q4(≥122)	*p*-Value	Q1(<48)	Q2(48–79)	Q3(79–109)	Q4(≥109)	*p*-Value
	*n* = 623	*n* = 606	*n* = 621	*n* = 613		*n* = 838	*n* = 851	*n* = 828	*n* = 858	
Age, years	49.2 (15.7)	52.6 (13.8)	55.6 (12.9)	56.6 (12.7)	<0.001	36.3 (9.9)	49.0 (10.4)	56.0 (10.9)	63.6 (10.5)	<0.001
BMI, Kg/m2	20.7 (7.3)	22.6 (2.8)	24.2 (2.6)	27.3 (3.8)	<0.001	20.6 (2.2)	22.4 (2.5)	24.2 (2.8)	26.7 (3.5)	<0.001
SBP, mm Hg	121.1 (16.3)	125.3 (16.0)	128.9 (17.5)	134.4 (18.5)	<0.001	109.8 (12.0)	120.4 (15.8)	127.6 (19.4)	136.8 (20.0)	<0.001
DBP, mm Hg	77.7 (10.4)	80.3 (10.2)	82.8 (10.6)	86.7 (10.8)	<0.001	72.4 (8.6)	77.8 (9.7)	80.8 (11.2)	83.8 (11.5)	<0.001
WC, cm	72.7 (5.9)	81.6 (3.4)	87.8 (3.3)	97.8 (5.8)	<0.001	72.1 (7.0)	78.8 (6.7)	84.0 (7.2)	92.0 (8.7)	<0.001
WHtR	0.45 (0.06)	0.49 (0.02)	0.53 (0.02)	0.58 (0.03)	<0.001	0.46 (0.04)	0.51 (0.04)	0.54 (0.04)	0.60 (0.06)	<0.001
WHR	0.83 (0.06)	0.88 (0.05)	0.91 (0.05)	0.95 (0.06)	<0.001	0.81 (0.06)	0.86 (0.10)	0.89 (0.10)	0.92 (0.26)	<0.001
TC mmol/L	4.6 (0.9)	4.8 (0.9)	4.9 (1.0)	5.1 (1.0)	<0.001	4.4 (0.9)	4.9 (1.0)	5.1 (1.0)	5.4 (1.0)	<0.001
Total triglycerides, mmol/L	0.9 (0.7–1.3)	1.2 (0.8–1.8)	1.5 (1.0–2.2)	2.1 (1.4–3.1)	<0.001	0.8 (0.6–1.1)	1.1 (0.8–1.6)	1.4 (1.0–1.9)	2.0 (1.4–2.8)	<0.001
HDL-C, mmol/L	1.6 (0.5)	1.4 (0.4)	1.3 (0.3)	1.2 (0.3)	<0.001	1.7 (0.6)	1.5 (0.4)	1.5 (0.3)	1.3 (0.3)	<0.001
LDL-C mmol/L	2.8 (1.1)	3.0 (0.8)	3.1 (1.0)	3.1 (1.0)	<0.001	2.6 (0.7)	3.1 (0.9)	3.3 (1.0)	3.3 (1.0)	<0.001
HbA1c, %	5.4 (0.8)	5.6 (0.9)	5.7 (1.0)	6.0 (1.1)	<0.001	5.3 (1.0)	5.5 (0.6)	5.7 (0.8)	6.0 (1.0)	<0.001
HbA1c, mmol/L	35.8 (8.5)	37.6 (9.3)	38.6 (10.5)	42.1 (11.7)	<0.001	34.4 (10.7)	36.3 (6.8)	38.7 (9.0)	42.4 (11.4)	<0.001
Glucose, mmol/L	5.2 (1.2)	5.4 (1.4)	5.6 (1.6)	6.1 (2.1)	<0.001	4.9 (0.6)	5.2 (0.9)	5.5 (1.4)	6.0 (1.8)	<0.001
Insulin, μU/mL	8.44(6.00–11.87)	9.18(6.44–13.30)	10.90 (7.64–15.09)	13.70 (9.58–20.58)	<0.001	8.98 (6.74–12.34)	10.10 (7.28–13.96)	10.87 (7.77–15.70)	13.53 (9.37–20.52)	<0.001
HOMA-IR	1.88 (1.27–2.66)	2.12 (1.42–3.11)	2.58 (1.74–3.78)	3.41 (2.28–5.60)	<0.001	1.97 (1.41–2.70)	2.27 (1.62–3.20)	2.53 (1.74–3.94)	3.36 (2.18–5.64)	<0.001
QUICKI	2.09 (1.99–2.20)	2.11 (2.00–2.22)	2.09 (2.00–2.21)	2.11 (2.01–2.26)	0.015	2.04 (1.95–2.15)	2.07 (1.98–2.17)	2.09 (2.00–2.19)	2.11 (2.01–2.24)	<0.001
hs-CRP, mg/L	1.0 (0.0–2.0)	1.0 (0.0–2.0)	1.0 (1.0–3.0)	2.0 (1.0–3.0)	<0.001	0.0 (0.0–1.0)	1.0 (0.0–2.0)	1.0 (1.0–3.0)	2.0 (1.0–4.0)	<0.001
Uric acid, mg/L	326.2 (89.2)	339.0 (118.7)	357.9 (116.3)	395.8 (119.3)	<0.001	230.7 (58.7)	249.2 (67.8)	271.3 (72.2)	314.4 (87.1)	<0.001
Current smoking	385 (61.8%)	343 (56.6%)	332 (53.5%)	304 (49.6%)	<0.001	5 (0.6%)	18 (2.1%)	29 (3.5%)	43 (5.0%)	<0.001
Current drinking	349 (56.0%)	358 (59.1%)	382 (61.5%)	359 (58.6%)	0.27	87 (10.4%)	87 (10.2%)	66 (8.0%)	74 (8.6%)	0.24
DM	21 (3.4%)	43 (7.1%)	58 (9.3%)	118 (19.2%)	<0.001	5 (0.6%)	22 (2.6%)	66 (8.0%)	149 (17.4%)	<0.001

BMI, body mass index; BP, blood pressure; WHtR, waist-to-height ratio; WHR, waist-to-hip ratio; HDL-C, high density lipoprotein cholesterol; LDL-C, low density lipoprotein cholesterol; HOMA-IR, homeostatic model assessment of insulin resistance; QUICKI, quantitative insulin sensitivity check index; hs-CRP, high sensitivity C reactive protein; DM, diabetes mellitus. Data are mean (SD) or median (interquartile range), unless otherwise stated.

**Table 2:** Characteristics of participants by body shape index (ABSI) among men and women in China (*n* = 5838).

	Men					Women				
	Q1	Q2	Q3	Q4	*p*-Value	Q1	Q2	Q3	Q4	*p*-Value
	*n* = 608	*n* = 544	*n* = 744	*n* = 567		*n* = 899	*n* = 892	*n *= 767	*n* = 817	
Age, years	49.3 (14.7)	51.8 (14.0)	53.9 (12.9)	59.1 (13.1)	<0.001	44.7 (13.5)	49.1 (13.2)	53.7 (12.8)	58.6 (14.4)	<0.001
BMI, Kg/m2	24.0 (8.4)	23.6 (3.4)	24.0 (3.3)	22.9 (3.5)	<0.001	23.3 (3.7)	23.7 (3.5)	24.0 (3.4)	23.1 (3.5)	<0.001
Systolic BP, mm Hg	124.2 (16.2)	127.0 (17.5)	129.0 (18.6)	129.2 (18.0)	<0.001	117.6 (16.3)	123.6 (20.1)	125.9 (19.9)	128.5 (21.0)	<0.001
Diastolic BP, mm Hg	80.4 (10.9)	81.9 (11.1)	82.5 (11.1)	82.5 (10.9)	0.002	76.3 (9.9)	79.4 (11.7)	79.7 (10.8)	79.8 (11.6)	<0.001
Waist circumference, cm	77.0 (9.3)	83.2 (8.3)	87.8 (8.5)	91.4 (9.3)	<0.001	73.5 (8.0)	80.2 (8.4)	84.6 (8.2)	89.8 (9.6)	<0.001
WHtR	0.47 (0.07)	0.50 (0.05)	0.53 (0.05)	0.55 (0.05)	<0.001	0.47 (0.05)	0.51 (0.05)	0.54 (0.05)	0.58 (0.06)	<0.001
WHR	0.84 (0.07)	0.88 (0.05)	0.91 (0.05)	0.95 (0.07)	<0.001	0.80 (0.06)	0.85 (0.07)	0.88 (0.05)	0.96 (0.28)	<0.001
CVAI	53.3 (41.7)	80.7 (39.4)	102.0 (40.2)	119.9 (42.0)	<0.001	54.0 (42.7)	73.8 (41.7)	89.2 (40.0)	99.4 (41.6)	<0.001
Total cholesterol, mmol/L	4.8 (1.0)	4.9 (0.9)	4.9 (1.0)	4.9 (1.0)	0.071	4.7 (1.0)	4.9 (1.0)	5.0 (1.0)	5.2 (1.1)	<0.001
Total triglycerides, mmol/L	1.2 (0.8–1.9)	1.2 (0.8–2.0)	1.4 (0.9–2.2)	1.4 (0.9–2.3)	<0.001	1.1 (0.7–1.6)	1.3 (0.8–1.9)	1.3 (0.9–2.1)	1.4 (0.9–2.1)	<0.001
HDL-cholesterol, mmol/L	1.4 (0.4)	1.4 (0.5)	1.4 (0.4)	1.4 (0.4)	0.17	1.5 (0.4)	1.5 (0.4)	1.5 (0.6)	1.5 (0.5)	0.14
LDL-cholesterol, mmol/L	2.9 (0.9)	3.0 (1.0)	3.0 (1.0)	3.0 (1.1)	0.38	2.9 (0.9)	3.0 (1.0)	3.1 (1.0)	3.2 (1.0)	<0.001
HbA1c, %	5.6 (0.9)	5.6 (0.8)	5.7 (0.9)	5.8 (1.1)	<0.001	5.5 (0.6)	5.6 (1.1)	5.7 (0.9)	5.8 (1.0)	<0.001
HbA1c, mmol/L	37.2 (9.5)	37.8 (8.5)	38.8 (10.1)	40.3 (12.6)	<0.001	36.1 (7.0)	37.4 (11.7)	38.7 (9.3)	39.9 (11.2)	<0.001
Glucose, mmol/L	5.4 (1.6)	5.5 (1.4)	5.7 (1.8)	5.7 (1.7)	<0.001	5.1 (0.9)	5.3 (1.1)	5.5 (1.3)	5.7 (1.9)	<0.001
Insulin, μU/mL	10.10 (6.95–14.07)	10.48 (6.97–14.99)	10.52 (7.33–15.14)	10.70 (7.14–15.90)	0.13	10.39 (7.66–14.61)	10.71 (7.58–15.41)	10.61 (7.85–16.15)	10.80 (7.58–15.81)	0.27
HOMA-IR	2.26 (1.50–3.35)	2.44 (1.55–3.68)	2.52 (1.66–3.87)	2.46 (1.63–4.15)	0.006	2.26 (1.64–3.39)	2.42 (1.68–3.69)	2.48 (1.73–3.89)	2.49 (1.65–4.01)	0.003
QUICKI	2.09 (1.98–2.19)	2.10 (2.00–2.22)	2.11 (2.02–2.23)	2.11 (2.00–2.24)	0.018	2.05 (1.96–2.15)	2.08 (1.99–2.17)	2.09 (2.00–2.20)	2.10 (2.01–2.23)	<0.001
hs-CRP, mg/L	1.0 (0.0–2.0)	1.0 (0.0–2.0)	1.0 (1.0–3.0)	1.0 (1.0–3.0)	<0.001	1.0 (0.0–2.0)	1.0 (0.0–2.0)	1.0 (1.0–3.0)	1.0 (1.0–3.0)	<0.001
Uric acid, mg/L	347.3 (127.8)	352.1 (114.8)	364.1 (112.0)	352.7 (100.7)	0.045	253.6 (73.5)	263.9 (78.3)	272.5 (80.3)	278.3 (81.2)	<0.001
Current smoking	320 (52.6%)	300 (55.1%)	429 (57.7%)	315 (55.6%)	0.33	19 (2.1%)	14 (1.6%)	26 (3.4%)	36 (4.4%)	0.002
Current drinking	343 (56.4%)	323 (59.4%)	453 (60.9%)	329 (58.0%)	0.39	77 (8.6%)	89 (10.0%)	73 (9.5%)	75 (9.2%)	0.77
DM	38 (6.3%)	43 (7.9%)	82 (11.0%)	77 (13.6%)	<0.001	31 (3.4%)	48 (5.4%)	67 (8.7%)	96 (11.8%)	<0.001

BMI, body mass index; BP, blood pressure; WHtR, waist-to-height ratio; WHR, waist-to=hip ratio; HDL-C, high density lipoprotein cholesterol; LDL-C, low density lipoprotein cholesterol; HOMA-IR, homeostatic model assessment of insulin resistance; QUICKI, quantitative insulin sensitivity check index; hs-CRP, hypersensitivity C reactive protein; DM, diabetes mellitus. Data are mean (SD) or median (interquartile range), unless otherwise stated.

**Table 3:** Correlations of CVAI and ABSI with metabolic variables among adults in China.

	CVAI (Age-and Sex Adjusted)		ABSI (Age-and Sex Adjusted)	
	*r*	*p*	*r*	*p*
Glucose	0.209	<0.001	0.042	0.002
HbA1c (%)	0.199	<0.001	0.044	<0.001
Insulin	0.133	<0.001	0.029	0.03
HOMA-IR	0.144	<0.001	0.035	0.008
TC	0.201	<0.001	0.034	0.009
TG	0.451	<0.001	0.054	<0.001
HDL-C	−0.420	<0.001	−0.005	<0.01
LDL-C	0.127	<0.001	0.022	0.09
Uric acid	0.292	<0.001	0.027	0.04
hs-CRP	0.021	0.11	0.012	0.35

HDL-C, high-density lipoprotein cholesterol; LDL-C, low-density lipoprotein cholesterol; HOMA-IR, homeostatic model assessment of insulin resistance; hs-CRP, high sensitivity C reactive protein.

**Table 4:** Adjusted odds ratios (ORs) and 95% CI of the visceral adiposity index and body shape index with diabetes risk in adults in China.

	Model 1	Model 2	Model 3	Model 4
Visceral adiposity index (CVAI)				
Men				
Q1	1	1	1	1
Q2	2.0 (1.2–3.5)	2.0 (1.2–3.5)	2.0 (1.1–3.4)	1.9 (1.1–3.3)
Q3	2.6 (1.5–4.3)	2.6 (1.5–4.3)	2.4 (1.4–4.0)	2.3 (1.4–3.9)
Q4	5.9 (3.6–9.6)	5.9 (3.6–9.5)	5.2 (3.1–8.5))	4.9 (2.9–8.1)
Women				
Q1	1	1	1	1
Q2	3.6 (1.3–9.7)	3.7 (1.4–9.9)	3.1 (1.2–8.4)	3.0 (1.1–8.2)
Q3	10.5 (4.1–27.1)	10.7 (4.1–27.5)	8.7 (3.2–21.7)	7.7 (2.9–21.0)
Q4	22.6 (8.7–58.9)	22.9 (8.8–59.9)	16.2 (6.1–43.2)	14.2 (5.3–38.2)
Body shape index (ABSI)				
Men				
Q1	1	1	1	1
Q2	1.2 (0.8–1.9)	1.3 (0.8–2.0)	1.2 (0.8–1.9)	1.2 (0.8–1.9)
Q3	1.7 (1.1–2.5)	1.8 (1.2–2.6)	1.6 (1.1–2.5)	1.6 (1.1–2.4)
Q4	1.9 (1.2–2.8)	1.9 (1.2–2.9)	1.9 (1.2–2.8)	1.8 (1.2–2.8)
Women				
Q1	1	1	1	1
Q2	1.3 (0.8–2.1)	1.4 (0.9–2.2)	1.2 (0.8–2.0)	1.2 (0.7–1.9)
Q3	1.9 (1.2–2.9)	1.2 (1.2–3.0)	1.8 (1.1–2.8)	1.7 (1.1–2.7)
Q4	2.0 (1.3–3.1)	2.1 (1.4–3.3)	2.0 (1.3–3.1)	2.0 (1.3–3.1)

Model 1: Adjusted for age; Model 2: Adjusted for age, residence, and smoking and alcohol use; Model 3: Adjusted for age, residence, smoking and alcohol use, total cholesterol, systolic BP and DBP; Model 4: Adjusted for age, residence, smoking and alcohol use, total cholesterol, systolic BP, DBP, uric acid and hypersensitive-C reactive protein.

**Table 5:** Sensitivity, specificity, Youden index and sex-specific cut-off points for various obesity indices in predicting diabetes risk among adults in China.

	Men				Women			
	Cut-off	Sensitivity (%)	Specificity (%)	Youden Index	Cut-off	Sensitivity (%)	Specificity (%)	Youden Index
CVAI	107.27	0.65	0.71	0.36	88.15	0.79	0.71	0.50
ABSI	0.08	0.72	0.57	0.28	0.08	0.84	0.57	0.41
BMI	24.73	0.86	0.46	0.32	23.18	0.82	0.63	0.45
WC	87.9	0.71	0.63	0.34	84.9	0.84	0.62	0.46

CVAI, Chinese visceral adiposity index; ABSI, body shape index; BMI, body mass index; WC, waist circumference.

## Supplementary Materials

The following are available online at https://www.mdpi.com/2072-6643/11/7/1580/s1, Figure S1: Flowchart of selection process for the data included in this analysis.
