# Supplementary material for: Comparisons of Visceral Adiposity Index, Body Shape Index, Body Mass Index and Waist Circumference and Their Associations with Diabetes Mellitus in Adults
Source: Nutrients. 2019 Jul 12;11(7):1580. doi: 10.3390/nu11071580 (PMC6683101; doi:10.3390/nu11071580)
Supplement: Supplementary file 1 [file nutrients-11-01580-s001.pdf]

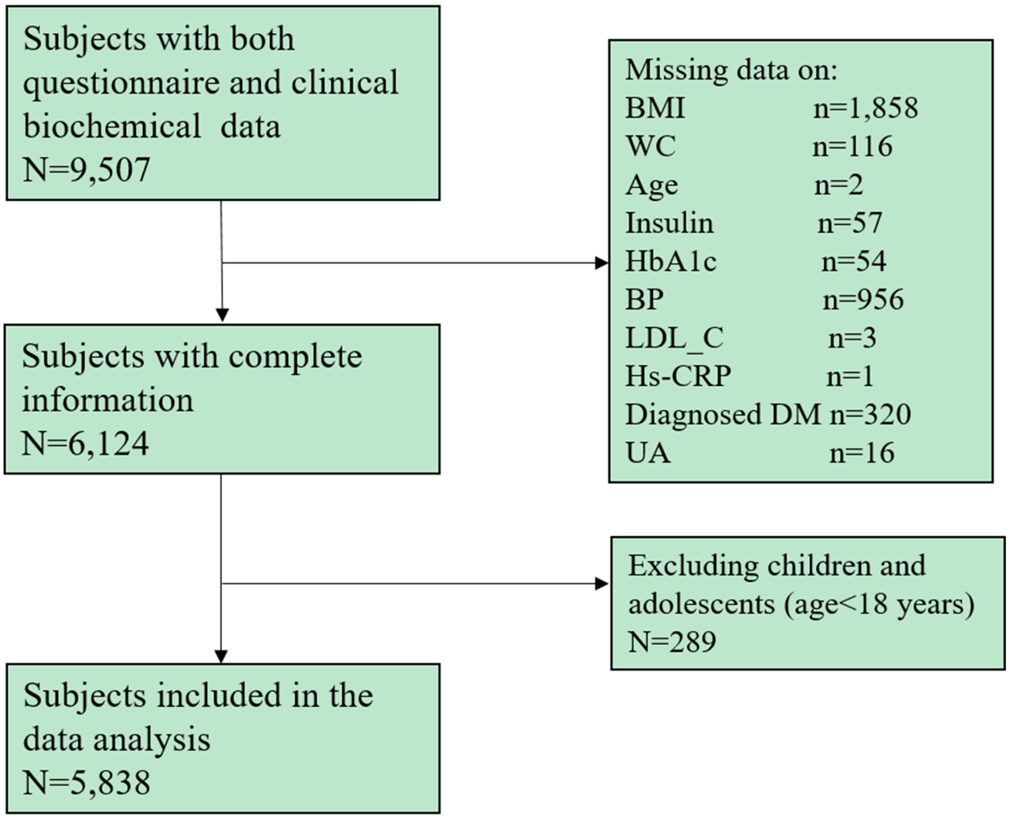

Supplementary Figure 1. Flowchart of selection process for the data included in this analysis

Abbr.: BMI, body mass index; WC, waist circumferences; BP, blood pressure; DM, diabetes mellitus; UA, uric acid; Hs-CRP, hypersensitive-C reactive protein; LDL-C, low-density lipoprotein cholesterol
